# Les critères diagnostiques et les particularités de prise en charge de l’angiomyolipome rénal: à propos de 8 cas

**DOI:** 10.11604/pamj.2016.25.182.7654

**Published:** 2016-11-22

**Authors:** Aziz El Majdoub, Abdelhak Khallouk, Moulay Hassan Farih

**Affiliations:** 1 Service d’Urologie, CHU Hassan II, Fès, Maroc

**Keywords:** Angiomyolipome, rein, sclérose tubéreuse de Bourneville, Angiomyolipoma, kidney, Bourneville’s tuberous sclerosis

## Abstract

L'angiomyolipome rénal est une tumeur rénale bénigne ayant une composante graisseuse. À travers une série de 8 cas nous montrons les critères diagnostiques et les particularités de prise en charges de l angiomyolipome rénal. Notre étude porte sur 8 cas d'angiomyolipomes rénaux durant une période de 4 ans, explorés par une échographie et une tomodensitométrie abdominales. le diagnostic a été retenu sur les données de l'imagerie dans tous les cas. Deux patients suivis pour une sclérose tubéreuse de Bourneville depuis l'enfance. L'âge moyenest de 42 ans. L'échographie aretrouvé un aspect hyperéchogène hétérogène dans tous les cas. La tomodensitométrie a permis la détection de la composante graisseuse. Le diagnostic radiologique de l'angiomyolipome rénal repose essentiellement sur la détection de la composante graisseuse. L'association à une sclérose tubéreuse de Bourneville est un argument diagnostique supplémentaire.

## Abstract (en)

Renal angiomyolipoma is a benign renal tumor with a fatty component. We here report a series of 8 cases in order to describe diagnostic criteria and management options for renal angiomyolipoma. We studied 8 cases of renal angiomyolipomas over a 4-year period. All patients underwent ultrasound and abdominal CT scan. The diagnosis was confirmed on the basis of imaging data in all the cases. Two patients had been treated for Bourneville’s tuberous sclerosis since childhood. The average age was 42 years. Ultrasound showed a hypoechoic heterogeneous aspect in all the cases. CT scan allowed the detection of the fatty component. Radiological diagnosis of renal angiomyolipoma is mainly based on the detection of the fatty component. Its association with Bourneville’s tuberous sclerosis is an additional diagnostic argument.

## Introduction

L'angiomyolipome (AML) est une tumeur bénigne rare dont le rein est le siège quasi-exclusif. Il représente 0,3% de l'ensemble des tumeurs du rein [1–3]. Il est souvent rencontré au cours des phacomatoses particulièrementla sclérose tubéreuse de Bourneville. Grâce aux différentes techniques d'imagerie, l'approche diagnostique est facilitée.

## Méthodes

C'est une étude rétrospective de 8 cas d'AML colligés dans notre service durant une période de 4 ans (2009 à 2012), explorés par une échographie abdominale et une tomodensitométrie abdominale sans et avec injection de produit de contraste (PDC) dans tous les cas. Le diagnostic d'angiomyolipome a été retenu sur les données del'imagerie, avec l'association avec une sclérose tubéreuse de Bourneville dans 2 cas.

## Résultats

**Figure 1:**
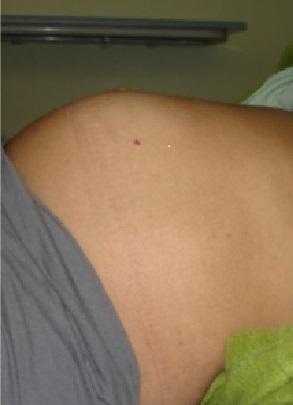
Image montrant une énorme voussure abdominale

**Figure 2:**
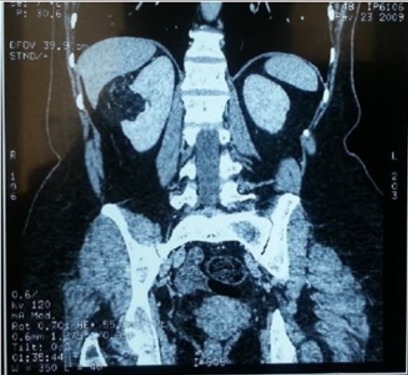
Image de reconstruction scannographique frontale montrant un angiomyolipome du rein droit

**Figure 3:**
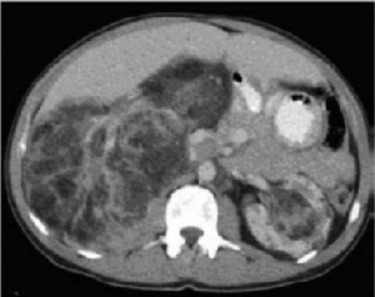
Image scannographique montrant un angiomyolipome du rein bilatéral

**Figure 4:**
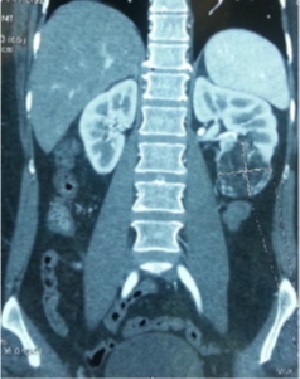
Image de reconstruction scannographique frontale montrant un angiomyolipome polaire inferieur du rein gauche

**Figure 5:**
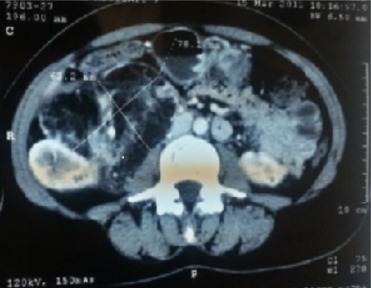
Coupe scannographique mettant en évidence un énorme angiomyolipone du rein droit

**Figure 6:**
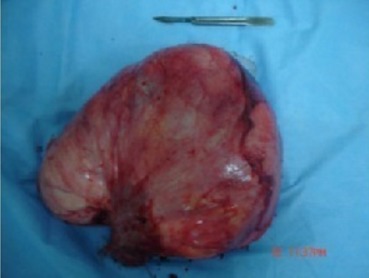
Aspect macroscopique de l angiomyolipome rénal

Nous avons recensé 6 femmes et 2 hommes dont l'âge moyen était de 42 ans avec des extrêmes allant de 17 à 53 ans. Dans 2 cas ces angiomyolipomes étaient intégrés dans la maladie de Bourneville. Le motif de consultation était une hématurie totale dans 4 cas, des douleurs lombaires dans 6 cas et une masse palpable dans 2 cas (Figure 1). Une découverte fortuite a été notée dans un seul cas, à l'occasion de la réalisation d'un angioscanner abdomino-pelvien dans le cadre du bilan étiologique d'une nécrose du gros orteil du pied droit. La localisation tumorale était bilatérale dans les 2 cas qui étaient tous atteints de maladie de Bourneville, unique dans 6 cas. Le rein droit était touché dans 4 cas, le rein gauche dans 2 cas. La taille des lésions variait entre 3,6 cm et 14,5 cm, avec une taille moyenne de 7 cm. L'échographie faite chez tous nos patients a retrouvé un aspect hyperéchogène hétérogène dans tous les cas. Aucune dilatation des voies excrétrices supérieures n'avait été rapportée. La tomodensitométrie réalisée chez les 8 patients a permis la détection de la composante graisseuse (Figure 2, Figure 3, Figure 4, Figure 5), Apres injection du produit de contraste, le rehaussement était intense dans deux cas, modéré dans trois cas et faible dans trois cas. La taille moyenne des lésions en TDM était de 7,66 cm avec des extrêmes de 3,6 à 14,55 cm. Aucun de nos patients n'avait présenté d'adénopathies profondes ni de lésion secondaire. Le traitement avait consisté à la réalisation de sept néphrectomies totales (Figure 6). L'abstention chirurgicale avec surveillance biologique de la créatinine plasmatique et radiologique par un uroscanner tous les 6 mois, était indiquée pour une patiente de 17 ans ayant un angiomyolipome bilatéral avec sclérose tubéreuse de Bourneville. Les principales indications de la néphrectomie totale dans notre série étaient : - la taille des tumeurs qui excédait à 4 cm exposant tous nos patients aurisque de rupture hémorragique. - incertitude concernant la bénignité de la tumeur. - angiomyolipomes symptomatiques (douleur, hématurie macroscopique) - destruction complète du parenchyme rénal pour deux patients. Toutes les néphrectomies ont été réalisées par voie d'abord antérieure transpéritonéale sous costal. Dans notre série, aucun décès lié à la chirurgie n'avait été observé, aucune transfusion pour hémorragie postopératoire n'avait été effectuée. Histologiquement, les prélèvements effectués au niveau des tumeurs montraient une prolifération tumorale bénigne faite de trois contingents dans tous les cas. Un contingent adipocytaire mature prédominant, un contingent de vaisseaux à paroi vasculaire épaisse et dystrophique ; le troisième était représenté par la prolifération de cellules musculaires qui semblaient naitre de la paroi vasculaire, ces cellules étaient fusiformes et avaient un noyau globuleux et vésiculeux. L'étude histologique n'avait pas objectivé d'atypies cytonucléaires ou de mitoses. Par contre des foyers nécrotiques étaient retrouvés dans deux prélèvements (observation 3 et 4), l'infiltration de la paroi de la veine rénale par la tumeur dans un autre cas (observation 5) et l'envahissement de la capsule et de la graisse périrénale dans l'un des deux cas présentant des remaniements nécrotiques Cela peut être en rapport soit avec l'expansion de l'AML soit en rapport avec un AML épithélioïde du rein. Mais le rapport anatomopathologique n'avait en aucun cas mis en évidence la présence de cellules épithélioïdes. Chez un seul patient, l'étude histologique avait mis en évidence une seconde tumeur associée à l'AML au même rein et correspondait au carcinome à cellules rénales. Il s'agissait d'un patient ayant la STB. L'étude immunohistochimique avait été réalisée pour tous les patients de notre série, elle avait permis la mise en évidence la positivité de l'anticorps anti HMB-45 dans tous les cas, confirmant ainsi le diagnostic d'AML rénal.

## Discussion

L'angiomyolipome est une tumeur relativement rare. Sa fréquence estestimée à 0,3% de l'ensemble des tumeurs rénales [1, 3]. Son incidence paraît augmenter en raison de sa découvertefréquente, de manière fortuite sur des examenséchographiques ou tomodensitométriques, ou sa recherchesystématique au cours de la sclérose tubéreuse deBourneville. 40 à 80% des patients atteints de cettephacomatose sont porteurs d'angiomyolipomes[1, 4, 5]. Dans notre série, 2 patients avaient une sclérose tubéreuse de Bourneville. Les angiomyolipomes isolés surviennent chez des sujets de la 5^ème^ décennie et touchent préférentiellement des femmes dans 50 à 80% des cas, alors que les angiomyolipomes associés à la sclérosetubéreuse de Bourneville atteignent des sujets plus jeunesau voisinage de la trentaine, répartis de façon égale entreles deux sexes [1, 3, 6]. Dans notre série, l'âge moyen était de 42 ans et le sexe féminin était retrouvé dans 6 cas. Les angiomyolipomes sont des tumeurs mésenchymateuses bénignesqui associent en proportion variable trois types de tissus:un tissu adipeux, un tissu musculaire et un tissu vasculaire. Ces tumeurs sont bien limitées quoique non encapsulées. Elles sont considérées comme des tumeurs bénignes sansaucun risque de récidive lorsque l'exérèse est complète, ni de métastases, ni de dégénérescence [2, 3]. L'angiomyolipome peutêtre associée à l'atteinte d'autres organes : ganglion, foie, rate. Cette atteinte extrarénale doit être interprétée commeétant l'une des manifestations multicentriques de cestumeurs et non comme des métastases [2, 3]. Les symptômes cliniques sont engendrés par leshémorragies intra-tumorales qui sont sources de douleurslombaires retrouvées dans 70 à 80% des cas, une pesanteur lombaire avec une masse palpable sont retrouvées dans 30 à 50% des cas, l'hématurie est retrouvée dans 30% des cas [1–3]. Nos patients ne présentaient aucune particularité clinique par rapport aux cas de la littérature.

L'abdomen sans préparation n'a plus actuellement d'intérêt diagnostique. Il peut montrer des calcifications volontierspériphériques, curvilignes, aspécifiques [3]. L'urographie intraveineuse montre en cas de tumeur rénalevolumineuse dépassant 3 cm de diamètre, un syndrome tumoral rénal non spécifique, avec une déformation des contours du rein et une déformation des cavité spyélocalicielles [1]. L'aspect échographique des angiomyolipomeest variable en fonctionde la taille de la tumeur. Le plus souvent, il se traduit, pour les tumeurs de petite taille, par un nodule hyperéchogène cortical, aussi échogène que le sinus rénal, homogène, à contour net et régulier [1, 3, 7]. Les angiomyolipomes volumineux sont généralement hétérogènes, comportant des plages hyperéchogènes, localisées ou diffuses [4]. Benchekroun [1] rapporte dans une étude de9 cas d'angiomyolipomes, un aspect hyperéchogène homogène chez 5 patients et hyperéchogène hétérogène chez 4 patients. Dans notre série, l'échographie faite chez les 8 patients a retrouvé un aspect hyperéchogène hétérogène avec une taille supérieure à 3 cm. L'aspect hyperéchogène est directement lié au contenu graisseux habituel de ces tumeurs et aux multiples interfaces désorganisées existant entre tissu graisseux et tissu non graisseux (vaisseaux sanguins et muscle lisse) [3, 6]. Ainsi, l'existence de multiples échostrès denses doit suggérer la présence de graisse intra-tumoraleet faire évoquer le diagnostic d'angiomyolipome [3]. Cet aspect hyperéchogène n'est toutefois pas spécifique et n'autorise en aucun cas un diagnostic de certitude. La confirmation est apportée par la tomodensitométrie par la mise en évidence de la composante graisseuse qui est un argument diagnostique majeur de ces tumeurs. La sensibilité de la TDM est de 90% dans le diagnostic des angiomyolipomes [7, 8]. Les composantes vasculaires etléiomyomateuse ont une densité tissulaire [3]. Après injection de produit de contraste, le contingent vasculairese rehausse très fortement, le contingent musculaire se rehausse peu. La mise en évidence d'une composante graisseuse intra-tumorale permet d'éliminer le diagnostic différentiel de cancer rénal. Celui-ci ne contient jamais de graisse [3, 6, 9]. Cette composante graisseuse a été notée dans tous les cas de notre série. Les limites du scanner sont représentées par les angiomyolipomes detaille inférieure à 15 mm [2], les hémorragies intra-tumoralesqui peuvent engendrer des modifications de l'aspect habituel masquant la densité du contingentgraisseux [1, 9]. L'IRM permet d'apporter des arguments diagnostiques supplémentaires en faveur de l'angiomyolipome rénal notamment dans la détection de la composante graisseuse qui se traduit par un signal intense homogène sur la séquence pondérée en T1, un signal modéré sur la séquence pondérée en T2 et suppression de ce dernier en technique de présaturation de la graisse. L'IRM trouve sa place également dans la mise en évidence des hémorragies intra-tumorales [1, 2]. L'angiomyolipome, bien que considéré comme un processus bénin, est susceptible d'avoir une extension péri-rénale, ganglionnaire ou endoveineuse rénale et cave inférieure[1, 4]. Cette extension est considérée comme une atteinte multifocale et non comme un processus métastatique [4]. L'IRM permet d'apporter des arguments diagnostiques supplémentaires en faveur de l'angiomyolipome rénal notamment dans la détection de la composante graisseuse qui se traduit par un signal intense homogène sur la séquence pondérée en T1, un signal modéré sur la séquence pondérée en T2 et suppression de ce dernier en technique de présaturation de la graisse. L'IRM trouve sa place également dans la mise en évidence des hémorragies intra-tumorales [1, 2]. Le traitement chirurgical de principe des volumineux angiomyolipomes rénaux, à haut risque hémorragique, doit être le plus conservateur possible. Le développement exo-rénal de ces lésions autorise souvent la réalisation d´une néphrectomie partielle avec résection cunéiforme de la tumeur [9, 10].

## Conclusion

Les angiomyolipomes sont des tumeurs bénignes rares. Le diagnostic radiologique repose essentiellement sur la détection de leur composante graisseuse. L'association à une sclérose tubéreuse de Bourneville est un argument diagnostique supplémentaire. L'évolution est marquée par les complications hémorragiques parfois graves.

### Etat des connaissances actuelle sur le sujet

- Le diagnostic radiologique repose essentiellement sur la détection de leur composante graisseuse;
- le risque de complications graves notament hémorragique justifie le traitement chirurgical;

des angiomyolipomes volumineux.

### Contribution de notre étude à la connaissance

- Apporter notre expérience à travers cette série de cas dans la prise en charge de l angiomyoliopme;
- Envahissement de la veine rénale dans un cas, ce qui est exceptionnel dans ce type de tumeur;
- L'angiomyolipome, bien que considéré comme un processus bénin, est susceptible d'avoir une extension péri-rénale, ganglionnaire ou endoveineuse rénale et cave inférieure. Cette extension est considérée comme une atteinte multifocale et non comme un processus métastatique.
